# Evaluating the relevance of ChatGPT in responding to clinical cases of treatment-resistant schizophrenia: a comparative analysis with clinical guidelines

**DOI:** 10.1192/j.eurpsy.2026.12120

**Published:** 2026-07-31

**Authors:** F. Zaouali, I. Anes

**Affiliations:** 1 Emergency department and mobile emergency and resuscitation service, Tahar Sfar University Hospital, Mahdia; 2 Outpatient psychiatric consultation, Hadj Ali Soua Regional Hospital, Ksar Hellal, Monastir, Tunisia

## Abstract

**Introduction:**

Treatment-resistant schizophrenia (TRS) remains a major therapeutic challenge in psychiatry, characterized by persistent psychotic symptoms despite adequate trials of antipsychotics. Recent advances in artificial intelligence (AI), especially large language models like ChatGPT, have sparked interest in their potential to assist clinicians by providing diagnostic and therapeutic suggestions. However, the accuracy and clinical appropriateness of AI-generated responses in complex psychiatric conditions such as TRS have not been thoroughly assessed.

**Objectives:**

To assess whether ChatGPT’s answers to clinical vignettes of TRS align with established evidence-based recommendations, and to determine the potential role of AI as a supplementary tool in psychiatric decision-making.

**Methods:**

We developed five detailed clinical vignettes representing common presentations of TRS based on DSM-5 criteria and typical treatment histories. Each case was input into ChatGPT (GPT-4) with prompts requesting diagnostic considerations, treatment options, and management strategies. ChatGPT’s responses were independently reviewed by two senior psychiatrists and compared to guidelines from the American Psychiatric Association (APA), the National Institute for Health and Care Excellence (NICE), and the World Federation of Societies of Biological Psychiatry (WFSBP). A qualitative content analysis was conducted to evaluate concordance with recommendations, presence of clinical nuance, and potential omissions or inaccuracies.

**Results:**

ChatGPT demonstrated a high degree of concordance with major guideline recommendations, accurately identifying TRS criteria, advocating clozapine initiation, and emphasizing psychosocial interventions. It also appropriately recommended monitoring for clozapine side effects and considering augmentation strategies when necessary. However, some responses lacked specificity regarding dose titration and did not consistently address non-pharmacological approaches in depth. Literature review revealed that while AI tools show promise in psychiatric education and preliminary assessments, limitations remain regarding complex decision-making and individualized care planning. Our findings suggest that ChatGPT can provide guideline-concordant, evidence-based suggestions for TRS cases but should be used as an adjunct rather than a substitute for clinical judgment.

**Conclusions:**

ChatGPT offers relevant and mostly guideline-compliant responses to clinical scenarios of treatment-resistant schizophrenia, supporting its potential as a supplementary tool in psychiatric practice. Nevertheless, limitations in contextualization and detailed management warrant cautious integration alongside expert clinical evaluation. Future research should focus on expanding AI capabilities for personalized psychiatry while maintaining rigorous validation against clinical standards.

**Disclosure of Interest:**

None Declared

